# The Treatment of Sore Nipples

**Published:** 1878-07-20

**Authors:** 


### THE TREATMENT OF SORE NIPPLES.

---

The measures recommended from ancient times to the present for the treatment of sore nipples are past enumeration, and yet there is nothing which may be employed with absolute certainty. On this account, the author takes occasion to report the following cases in which he has used carbolic acid "with so direct and striking results" that he deems it his duty to recommend this agent in the affection under consideration:

1. Frau S., confined for the first time in August, 1876. On the third day, she presented upon the right nipple several fissures and two vesicles which were very painful upon nursing the child. The fissures were cauterized, and the vesicles opened with the nitrate of silver pencil. After lead-water applications were made, and less often application of the child to the affected side was enjoined. In spite of this careful, thorough treatment, two painful weeks passed away before the nipple was completely healed.

Early in October, 1877, the woman was delivered the second time. She must have suspended nursing after about five months, on account of the new conception. This time, she showed, on the third day after birth, upon both nipples, several vesicles, as well as several fissures. The vesicles were emptied, and all sore places were again touched with the nitrate of silver pencil. The pain, upon the application of the child, was diminished so little that, in the evening, an attempt was made to protect the breast with a rubber nipple shield, but this was rejected. The next day, the fissures were enlarged, very red, and, like the borders of the vesicles, bleeding easily. An experiment with carbolic acid was now determined upon. Applications of a five per cent. solution were employed, which were renewed every two or three hours. Of course, before every nursing, the breast was carefully cleansed in order to prevent any taking up of the acid by the child.

The solution was tepid; a clean linen cloth was moistened with it and laid upon the nipple. Directly after the first application, the pain abated, and it did not increase when the child was put to the previously carefully cleansed part. The next day, the fissures were smaller and paler, the eschar gone, and, after two days' longer use of the solution, healing was complete. No more pain was felt in the breast from nursing.

2. Several weeks after the above, a lying-in woman presented, both of whose nipples were sore. The nipples were well projected and of normal size. Upon the surface of the right one, there were two fissures, and upon the left one, one fissure, about four mm. long and two mm. deep, the left arising from a burst vesicle, with a superficial eschar upon the skin of the nipples. This was treated in the same manner, but with only a two per cent. solution renewed every two hours. After, probably, two hours, the child was again applied; the left nipple manifested far less sensibility, but the right one was yet painful. The application of the solution was continued. Although it was not made regularly the next night, the sucking and grasping of the child of the left nipple were entirely painless. The right one, on the contrary, had been much more painful since the day before. Upon inspection, the fissures were found decreased in size and paler. Notwithstanding the irregular employment of the solution, the pain was eased in the right nipple during the course of the day, and the woman was able to nurse without the least annoyance. Eight days afterward, there was experienced a new pain of violent character in the right nipple, which was later ascertained to proceed from a small recently formed fissure. One application of a five per cent. solution of carbolic acid was made. At first, it excited somewhat severe smarting, but it sufficed to render completely painless the next nursing of the child, and in two days, the fissure had disappeared.—*Ex.*

